# Response to Immunotherapy in Adenocarcinoma Lung With Gastric Metastasis: A Rare Case Report and Review of Literature

**DOI:** 10.7759/cureus.19790

**Published:** 2021-11-21

**Authors:** Saroj Kumar Das Majumdar, Bikash Ranjan Mahapatra, Anupam Muraleedharan, Dillip Kumar Parida, Amit Kumar Adhya

**Affiliations:** 1 Radiation Oncology, All India Institute of Medical Sciences, Bhubaneswar, IND; 2 Pathology and Lab Medicine, All India Institute of Medical Sciences, Bhubaneswar, IND

**Keywords:** pd-l1, pembrolizumab, immunotherapy, lung cancer, gastric metastasis

## Abstract

Worldwide lung cancer is the most common cause of cancer mortality. Most of the lung cancer patients present with advanced disease at the time of diagnosis, and in that case, the prognosis is poor even with treatment. The most common sites of metastases in non-small-cell lung cancer are the brain, bone, liver, and adrenal gland. Metastasis to the stomach is extremely rare, which carries with itself a more dismal prognosis. Here we are reporting a rare case of adenocarcinoma lung with metastasis to the stomach, which was initially a diagnostic dilemma. The patient survived for 30 months from the diagnosis of gastric metastasis by management predominantly with immunotherapy.

## Introduction

Worldwide, lung cancer is the leading cause of cancer mortality. It is also the second most common malignancy in both sexes (11.4%) after breast cancer (11.7%) [1]. Most of the patients at presentation have an advanced disease, which leads to an overall poor outcome [2]. Non-small-cell lung cancer is much more common than small-cell lung cancer. Among non-small-cell lung cancers, adenocarcinoma is the predominant histopathology [3,4]. Stage IV patients with chemotherapy have survival of less than one year, while targeted therapy and immunotherapy further increase survival [5-8]. The most common sites of metastases in non-small-cell lung cancer are the brain, bone, liver, adrenal, and lung. Metastasis to the stomach is extremely rare in lung cancer and there is very little literature regarding the same. But the incidence of lung metastasis in carcinoma stomach is not uncommon, which leads to bias in clinical decision-making and its treatment.

Here we report a case of adenocarcinoma lung with metastasis to the stomach and review of the literature.

## Case presentation

**Figure 1:**
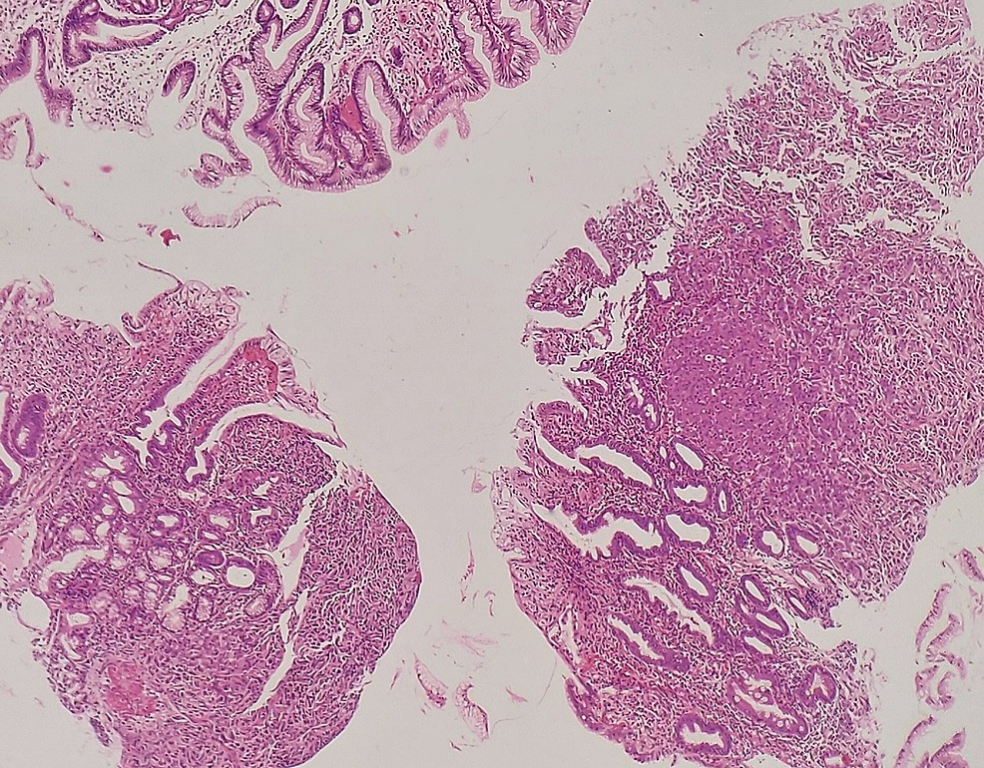
Gastric biopsy that showed a diffusely infiltrating tumor in the lamina propria. H&E stain, 100x. H&E, hematoxylin and eosin.

**Figure 2:**
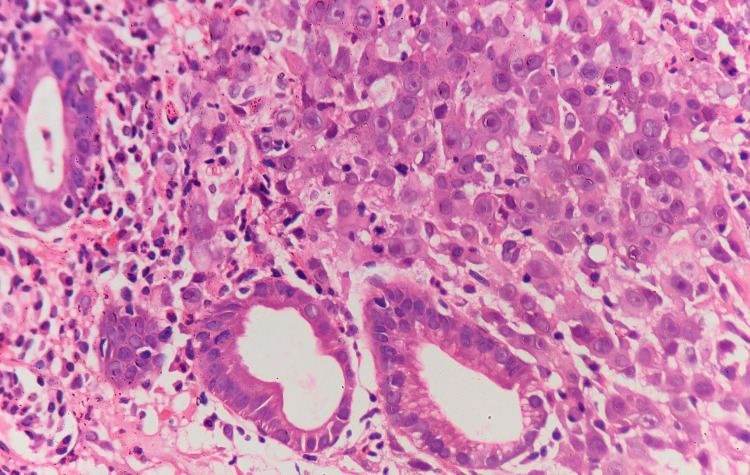
Gastric biopsy showing tumor cells which are large, with vesicular nuclei and prominent nucleoli. No glandular or mucinous differentiation is noted. H&E stain, 400x. H&E, hematoxylin and eosin.

**Figure 3:**
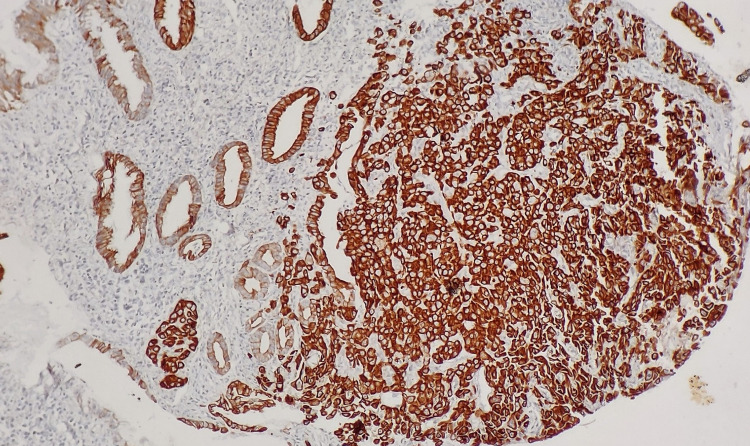
Tumor cells from gastric biopsy show strong immunopositivity for CK-7. CK-7, cytokeratin 7.

**Figure 4:**
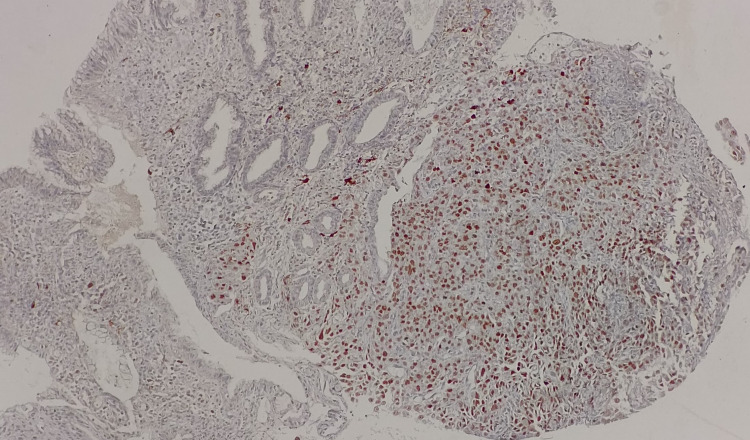
Tumor cells from gastric biopsy show strong immunopositivity for TTF-1. TTF-1, thyroid transcription factor 1.

A 72-year-old male, known hypertensive on medication, non-smoker, no family history of cancer presented with complaints of pain in the right hip with difficulty in walking in January 2019. On evaluation, a pathological fracture was found at the neck of the right femur. Magnetic resonance imaging of the spine was done, which revealed osteophyte complexes at C3-C4, C4-C5, C5-C6 vertebrae causing narrowing of neural foramina. Multiple T2-hyperintense lesions in lung parenchyma were an incidental finding. Upon further evaluation with positron emission tomography-computed tomography (PET-CT) scan of the whole body, mass in the apex of the right lung, right hilum, mediastinal lymph node, soft tissue wall thickening in the proximal stomach along with multiple liver and bone metastases were found. Upper gastrointestinal (GI) endoscopy revealed a proximal gastric growth from which a biopsy was taken. Histopathology showed poorly differentiated adenocarcinoma. A provisional diagnosis of carcinoma stomach with distant metastasis was reached. But immunohistochemistry came out to be positive for thyroid transcription factor-1 (TTF-1) and cytokeratin-7 (CK-7), while negative for cytokeratin 20 (CK-20) (Figures 1-4).

So, a possibility of metastasis from a lung primary was considered. It was confirmed by a biopsy from the lung mass that revealed adenocarcinoma as the histopathology. On immunohistochemistry, it was positive for TTF-1 and CK-7 while negative for CK-20 and synaptophysin. Analysis for anaplastic lymphoma kinase, epidermal growth factor receptor, and receptor tyrosine kinase 1 were all negative but programmed death ligand 1 (PD-L1) tumor proportion score (TPS) was 90%. So, the final diagnosis was adenocarcinoma lung with multiple lung, liver, femur, as well as gastric metastases.

**Figure 5:**
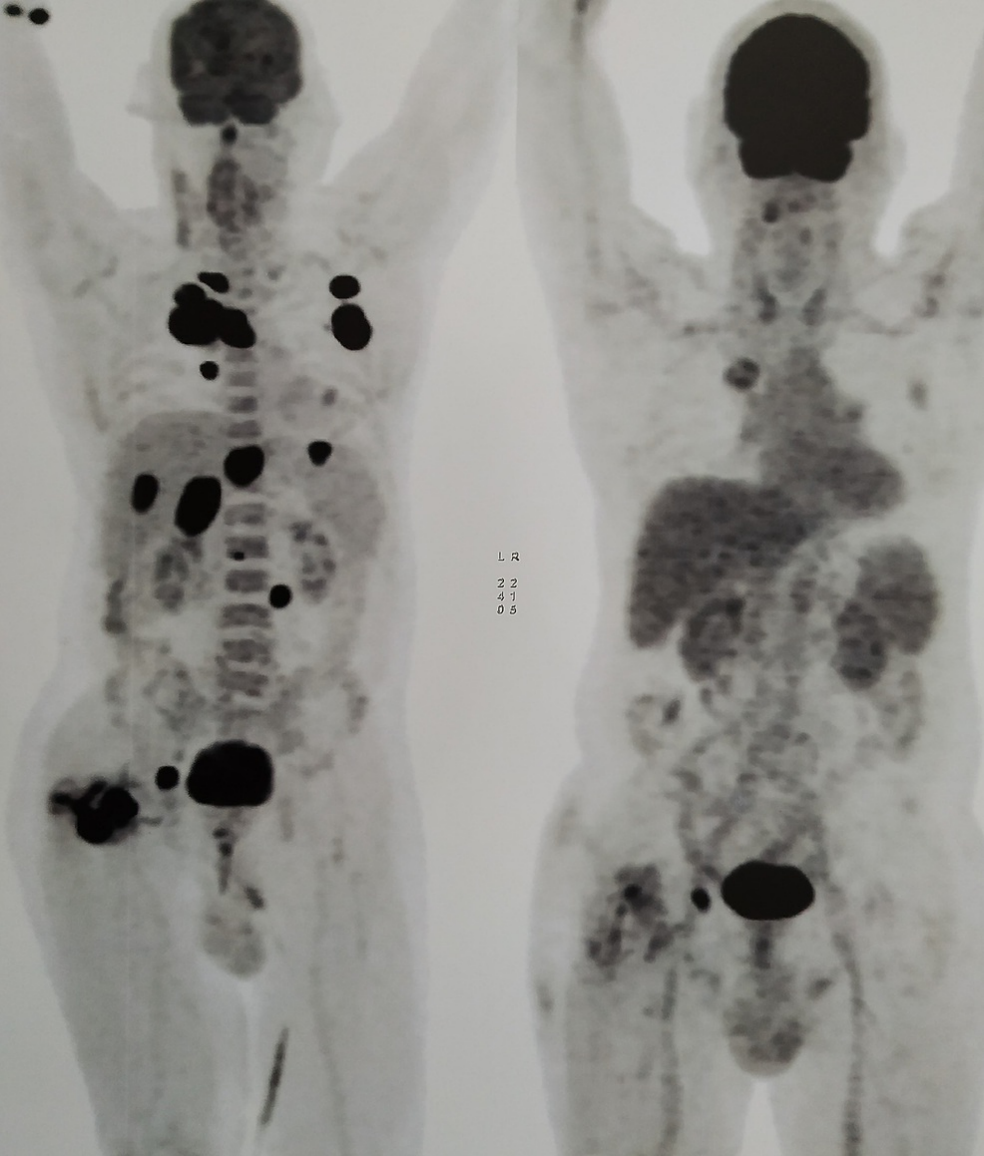
Interim whole-body PET-CT scan after four cycles of pembrolizumab showing near-complete response compared to baseline PET-CT. PET-CT, positron emission tomography-computed tomography.

**Figure 6:**
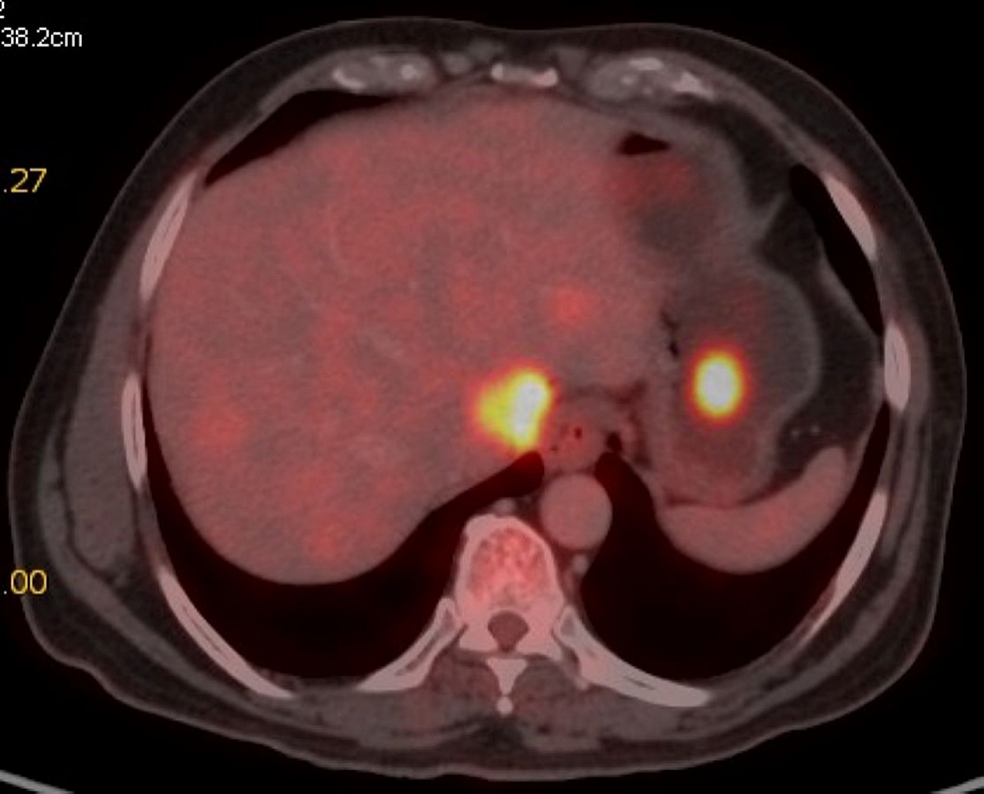
Baseline PET-CT scan showing lesions in liver (size 2.8 x 2.9 cm, SUVmax = 23.07) and stomach (size 1.9 x 2.1 cm, SUVmax = 13.05). PET-CT, positron emission tomography-computed tomography; FDG, fluorodeoxyglucose; SUVmax, maximum standardized uptake value.

**Figure 7:**
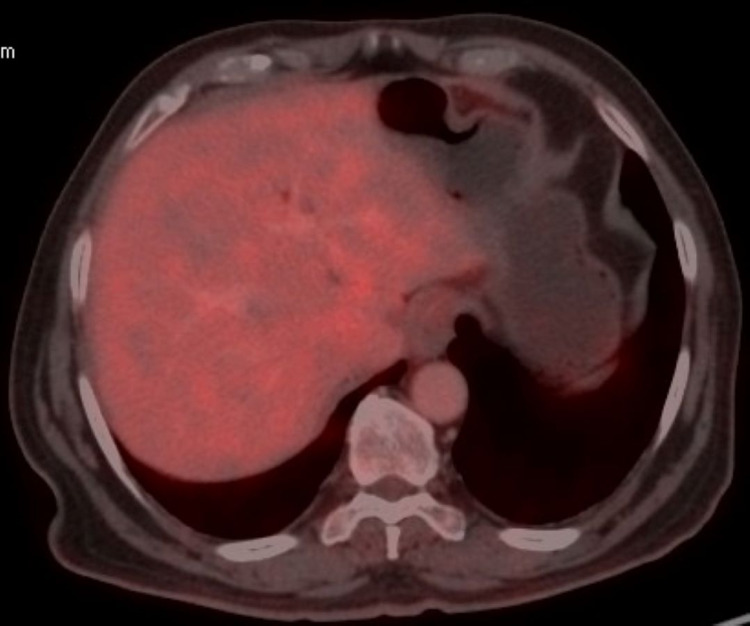
PET-CT scan after four cycles of pembrolizumab showing complete metabolic response in liver and stomach. PET-CT, positron emission tomography-computed tomography.

For the pathological fracture, he underwent fixation by intramedullary nailing followed by palliative external beam radiotherapy 8 Gy in a single fraction. He received 10 three-weekly cycles each of Inj. pembrolizumab 200 mg as intravenous infusion and Inj. denosumab 120 mg subcutaneously from March 2019 to September 2019. An interim PET-CT scan done after four cycles of immunotherapy in June 2019 showed a near-complete metabolic response (Figures 5-7).

**Figure 8:**
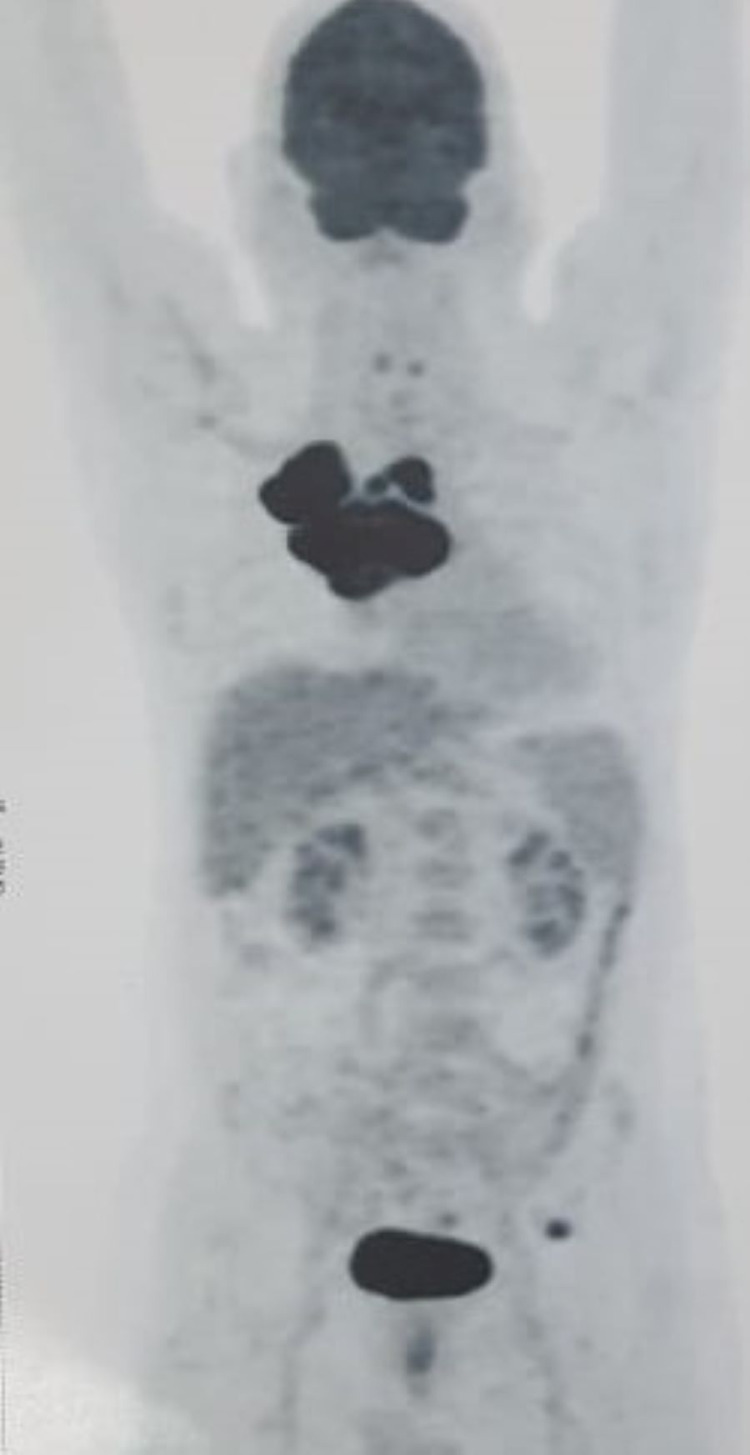
Whole-body PET-CT scan showing significant interval increase in metabolic activity at primary site. PET-CT, positron emission tomography-computed tomography.

**Figure 9:**
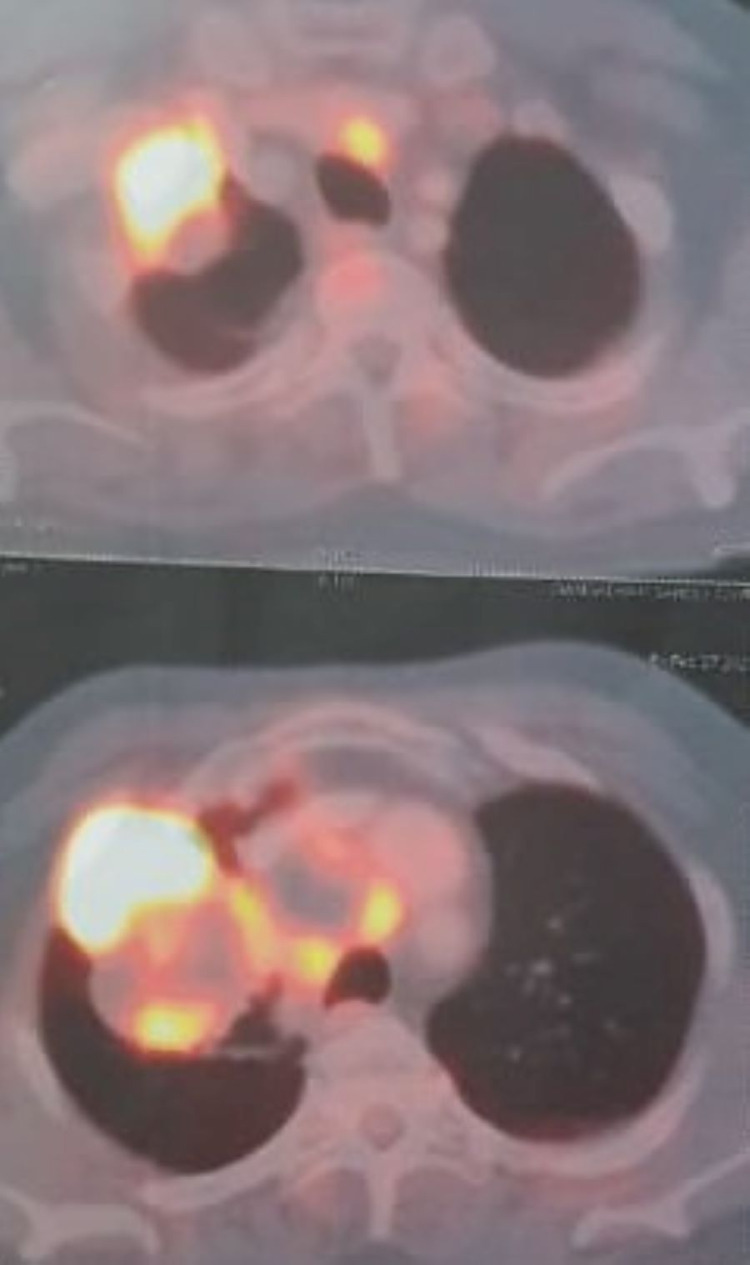
Re-evaluation PET-CT scan in February 2021 showing lesion (size 6.4 x 5.4 x 7.2 cm, SUVmax = 37.04) in upper lobe of right lung. PET-CT, positron emission tomography-computed tomography; SUVmax, maximum standardized uptake value.

After 10 cycles, Inj. pembrolizumab was discontinued as the patient was unwilling to continue due to personal reasons. He was then started with chemotherapy, Inj. pemetrexed 500 mg/m^2^ and Inj. bevacizumab 15 mg/kg in October 2019. Following this, the patient developed grade 1 maculopapular rash, pedal edema, and grade 2 diarrhea, which were managed conservatively. After that the patient did not opt for the continuation of chemotherapy. The next visit of the patient was in February 2021 with complaints of cough and breathlessness. On re-evaluation with PET-CT scan, progressive disease was found in the primary site (Figures 8, 9).

**Figure 10:**
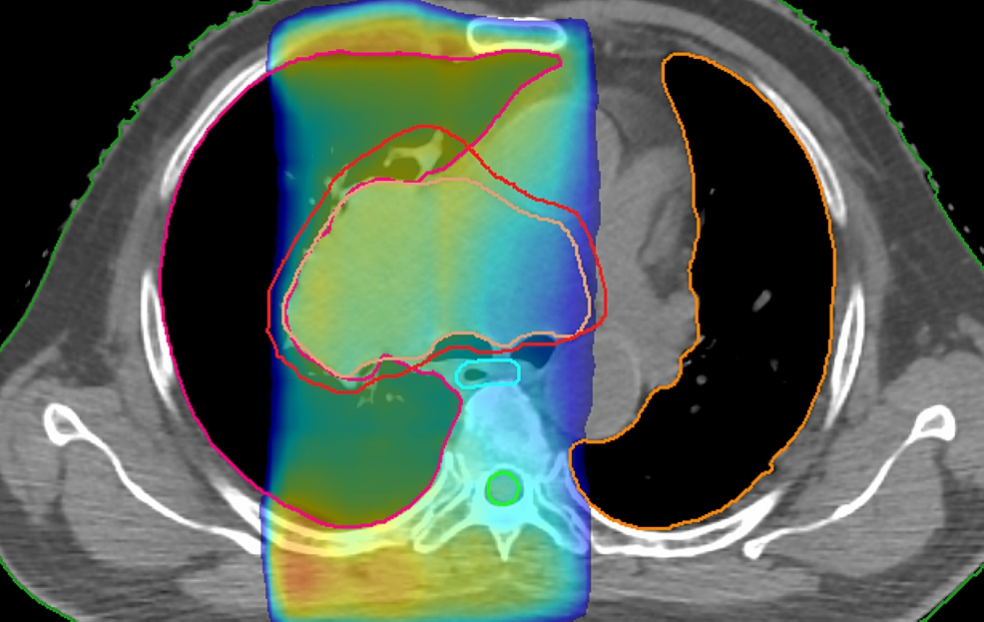
3D-CRT plan for palliative radiotherapy to primary disease with color wash showing 95% isodose coverage. 3D-CRT, three-dimensional conformal radiotherapy.

Palliative radiotherapy of 30 Gy in 10 fractions to the primary lesion was delivered through anteroposterior and posteroanterior portals of 10 megavolt energy each in April 2021 via linear accelerator (Elekta-Versa HD) which was tolerated well (Figure 10).

In June, the patient developed an altered sensorium and contrast-enhanced magnetic resonance imaging of brain revealed multiple brain metastases for which the patient received palliative whole-brain radiotherapy of 30 Gy in 10 fractions over two weeks from the same linear accelerator. His condition further deteriorated, and in July, the patient succumbed to disease progression.

## Discussion

In India, lung cancer is the fourth most common malignancy in incidence as well as mortality while stomach cancer stands at sixth position [9]. The most common sites of metastasis in lung cancer are the brain, bone, liver, adrenal, and lung, while that in stomach cancer are liver, peritoneum, lung, and bone [10]. In the context of malignancy, if the disease involves both stomach and lungs, the first impression is that of a stomach cancer metastasizing to the lung. Primary malignancy of lung with metastasis to stomach is extremely rare (<2%) [11,12]. Also, metastasis to the stomach usually occurs from the breast, lung, esophagus, kidney, and melanoma [13]. In terms of histology, metastasis to the stomach from a lung primary is more common with squamous cell carcinoma lung [14]. This rare case is that of adenocarcinoma, which further compels toward a provisional diagnosis of stomach primary.

The clinical presentation of gastric metastasis is indistinguishable from a primary gastric cancer with symptoms like melena, anemia, epigastric pain, nausea, and vomiting [13]. Upper GI endoscopy findings may help in differentiating gastric metastasis from the primary tumor, but histopathology and immunohistochemistry are necessary for a definitive diagnosis. In our case, there were no symptoms related to the gastric lesion and was an incidental finding in the PET-CT scan. On immunohistochemistry, CK-7 and TTF-1 were positive and CK-20 was negative. CK-7 positivity in lung adenocarcinoma is 100% while that in stomach adenocarcinoma is 38%. CK-20 positivity in lung adenocarcinoma is 10% while in the stomach it is 50% [15].

Survival in stage IV lung cancer without any driver mutation is approximately 10-12 months with palliative chemotherapy [5]. With the use of targeted therapy in patients harboring driver mutations, the median overall survival improves by approximately 25 months. The use of immunotherapy increases overall survival further [16]. The five-year overall survival rate exceeded 25% among patients with a PD-L1 TPS of 50% or greater. Also, the tumors having a high PD-L1 score show a favorable response to immunotherapy [17]. In general, the outcome after metastasis to the stomach has been uniformly poor, with the overall survival ranging from three months to one year.

In the case of lung cancer with metastasis to the stomach, all the previous case studies had used palliative chemotherapy and/or best supportive care [12,18,19]. To the best of our knowledge, this is the first study with experience of immunotherapy in this setting. It is noteworthy that the patient developed a complete response in PET-CT scan after four cycles of immunotherapy, which may be due to high PD-L1 expression which is again reflected as a sustained response even after discontinuation of pembrolizumab and a survival period of 30 months from the time of diagnosis of stomach metastasis. It was also found that immunotherapy was well tolerated with minimal toxicity.

## Conclusions

Adenocarcinoma of lung metastasizing to stomach is a rare finding with poor outcome. Immunohistochemistry and PET-CT scan are crucial for the diagnosis. Multiple treatments like palliative chemotherapy, immunotherapy, and radiotherapy can be given as per the clinical situation. Immunotherapy can significantly improve survival and expression of PD-L1 is a predictor of response to immunotherapy.
